# Commentary: The ubiquitin-proteasome system in the tumor immune microenvironment: a key force in combination therapy

**DOI:** 10.3389/fimmu.2025.1522427

**Published:** 2025-03-27

**Authors:** Lu Zhang, Liping Jia, Linhan Wang, Wenxiu Yuan, Xin Shi

**Affiliations:** ^1^ Qingdao Hospital, University of Health and Rehabilitation Sciences (Qingdao Municipal Hospital), Qingdao, China; ^2^ Department of Life Science, Imperial College London, London, United Kingdom

**Keywords:** ubiquitin-proteasome system, tumor immune microenvironment, proteolysis targeting chimeras (PROTACs), Foxp 3, ubiquitination, cancer therapy

## Introduction

The review by Yongmei Wang et al., “The ubiquitin-proteasome system in the tumor immune microenvironment: a key force in combination therapy,” provides a comprehensive analysis of the UPS’s role in the TIME, highlighting its potential as a therapeutic target in immunotherapy (1). Despite this, the review could benefit from a more detailed discussion on the UPS’s impact on the transcription factor FOXP3, the regulation of immune cell function by E3 ubiquitin-protein ligases, and the emerging role of Proteolysis Targeting Chimeras (PROTACs) in modulating E3 ligase activity.

## FOXP3 and Treg biology

A significant aspect that requires emphasis is the role of FOXP3 in Tregs, which is crucial for maintaining immune homeostasis and suppressing excessive immune responses. The stability of FOXP3 is vital for the immunosuppressive function of Tregs, and its dysregulation can lead to severe immune deficiencies or contribute to tumor immune evasion. Overexpression of FOXP3 results in an abnormal increase in Tregs, which can be detrimental to effective immune responses against tumors. The review should highlight the post-translational modifications, particularly ubiquitination, that regulate FOXP3 stability and function (2).

## Regulation of FOXP3 by E3 ligases

The activity and expression of FOXP3 are regulated by various E3 ubiquitin-protein ligases, such as CBLB and STUB1. These ligases directly affect FOXP3 levels in Tregs, with implications for immunosuppression within the TIME. For instance, CBLB has been shown to negatively correlate with FOXP3 expression, suggesting a role in modulating Treg function. STUB1 overexpression in Tregs leads to decreased FOXP3 protein levels and a weakening of Tregs’ immunosuppressive function. Different FOXP3 E3 ubiquitin ligases may affect the expression of FOXP3 through different mechanisms. Itch promotes the nuclear translocation and transcriptional activity of FOXP3 through K63-linked ubiquitination modification (a non-degradative type) (3). In Itch-deficient mice, the nuclear localization of FOXP3 in Treg cells is reduced by 50%, leading to an autoimmune phenotype. The knockout of RNF20 decreases the mRNA level of Foxp3 in Treg cells by 70%; TRAF6 catalyzes the K63 ubiquitination of FOXP3 at Lys227, promoting the binding and stabilization of the deubiquitinase USP7 (4); RNF31 catalyzes the M1-linked linear ubiquitination of FOXP3, antagonizing the K48 degradation signal (5). The RNF31 inhibitor HOIPIN-8 reduces the FOXP3 level in Treg cells by 60%, enhancing the anti-tumor immunity. As a component of the CRL2^(KLHDC2) complex, KLHDC2 mediates the K48-linked ubiquitination degradation of FOXP3 (6). The knockout of KLHDC2 extends the half-life of FOXP3 by 4 times and enhances the inhibitory function of Treg cells by 2.3 times.

The UPS influences Treg biology or immunology was important as well, for example the USP5 inhibition alongside PD-(L)1 blockade as a promising cancer treatment strategy (7). Identifying E3 ligases and their mechanisms in cancers like breast cancer can provide new therapeutic targets for patients, especially those with advanced, recurrent, and metastatic disease (8).

In terms of drug development, there has been significant progress in targeting the UPS for cancer therapy. Proteasome inhibitors, such as bortezomib, have been approved for the treatment of multiple myeloma and mantle cell lymphoma (9). Additionally, the development of deubiquitinating enzyme (DUB) inhibitors is an emerging area of research, with several compounds entering preclinical and clinical trials. For instance, the USP7 inhibitor P5091 has shown promise in the treatment of multiple myeloma by promoting the ubiquitination and degradation of MDM2, thereby activating the p53 pathway (10).

The review also provides a thorough summary of the current research on the potential of E3 ligases as drug targets. E3 ligases, such as SPOP and FBXO22, have been shown to ubiquitinate and degrade PD-L1, suggesting that their inhibition could enhance anti-tumor immune responses (11). Furthermore, the role of neddylation in regulating the activity of CRL E3 ligase complexes has been highlighted, with the potential for neddylation inhibitors to disrupt TAM recruitment and tumor immune evasion (12).

## Proteolysis targeting chimeras

Proteolysis Targeting Chimeras (PROTACs) is a promising therapeutic strategy in immunotherapy (13), with one end targeting the protein of interest (POI) and the other end binding to a ubiquitin E3 ligase. By bringing POI and E3 into close proximity, POI is ubiquitinated by E3 and subsequently degraded by the proteasome. This technology primarily recruits E3 by utilizing small molecules that specifically bind to E3. While there are over 600 E3 ligases encoded by the human genome, a limited number have well-defined targeting small molecules suitable for PROTAC technology. Identifying E3 ligases and their mechanisms in breast cancer can provide new therapeutic targets for patients, especially those with advanced, recurrent, and metastatic disease. KLHDC2 is the efficient E3 degrader for FOXP3, and identification of small-molecule PROTAC targeting KLHDC2 and FOXP3 is a potential strategy for immunotherapy.

Besides, the E3 ligases can regulate immune cells and the immune microenvironment through epigenetic mechanisms, leading to the loss of H2AK119 monoubiquitination and the loss of H3K27 trimethylation(such as CUL4B), which results in the depression of target genes closely associated with cell growth and migration. Therefore, the selection of E3 as a target should focus on specific structural domains for different target proteins in different genetic landscapes (1, 12, 13).

## Combination therapies

The potential synergistic effects of combining UPS-targeted therapies with other immunotherapeutic strategies, such as immune checkpoint inhibitors, should be explored.The review could benefit from discussing preclinical data and correlating it with clinical outcomes to highlight the translational potential of targeting the UPS in cancer immunotherapy.

A more detailed analysis of the immunosuppressive network within the TIME, including the interplay between Tregs, myeloid-derived suppressor cells (MDSCs), and tumor-associated macrophages (TAMs), would provide a more comprehensive view of the immunological landscape.

In conclusion, while the review by Wang et al. provides a solid foundation for understanding the UPS’s role in the TIME, it would be enhanced by incorporating the above suggestions. By emphasizing the role of E3 ligases in regulating immune cell function and the therapeutic potential of PROTACs, the review could provide a more holistic view of the UPS’s impact on immune cell function and its potential as a therapeutic target in cancer immunotherapy.

## References

[1] Wang Y Li S Wang W. The ubiquitin-proteasome system in the tumor immune microenvironment: a key force in combination therapy. Front Immunol. 15:1436174. doi: 10.3389/fimmu.2024.1436174 PMC11416925 39315102

[2] Miyara M Yoshioka Y Kitoh A Shima T Wing K Niwa A. Functional delineation and differentiation dynamics of human CD4+ T cells expressing the FoxP3 transcription factor. Immunity. (2009) 30:899–911. doi: 10.1016/j.immuni.2009.04.009 19464196

[3] Venuprasad K Huang H Harada Y Elly C Subramaniam M Spelsberg T Su J. The E3 ubiquitin ligase Itch regulates expression of transcription factor Foxp3 and airway inflammation by enhancing the function of transcription factor TIEG1. Nat Immunol. (2008) 9:245–53. doi: 10.1038/ni1564 PMC2610020 18278048

[4] Ni X Kou W Gu J Wei P Wu X Peng H. TRAF6 directs FOXP3 localization and facilitates regulatory T-cell function through K63-linked ubiquitination. Embo j. (2019) 38. doi: 10.15252/embj.201899766 PMC6484404 30886050

[5] Zhu F Yi G Liu X Zhu F Zhao A Wang A. Ring finger protein 31-mediated atypical ubiquitination stabilizes forkhead box P3 and thereby stimulates regulatory T-cell function. J Biol Chem. (2018) 293:20099–111. doi: 10.1074/jbc.RA118.005802 PMC6311505 30389786

[6] Röth S Kocaturk NM Sathyamurthi PS Carton B Watt M Macartney TJ. Identification of KLHDC2 as an efficient proximity-induced degrader of K-RAS, STK33, β-catenin, and FoxP3. Cell Chem Biol. (2023) 30:1261–76.e7. doi: 10.1016/j.chembiol.2023.07.006 37591251

[7] Shao N Xi L Lv Y Idris M Zhang L Cao Y. USP5 stabilizes YTHDF1 to control cancer immune surveillance through mTORC1-mediated phosphorylation. Nat Commun. (2025) 16:1313. doi: 10.1038/s41467-025-56564-9 39900921 PMC11791202

[8] Békés M Langley DR Crews CM. PROTAC targeted protein degraders: the past is prologue. Nat Rev Drug Discov. (2022) 21:181–200. doi: 10.1038/s41573-021-00371-6 PMC8765495 35042991

[9] Li X Pu W Zheng Q Ai M Chen S Peng Y. Proteolysis-targeting chimeras (PROTACs) in cancer therapy. Mol Cancer. (2022) 21:99. doi: 10.1186/s12943-021-01434-3 35410300 PMC8996410

[10] Chauhan D Tian Z Nicholson B Kumar KGS Zhou B Carrasco R. A Small Molecule Inhibitor of Ubiquitin-Specific Protease-7 Induces Apoptosis in Multiple Myeloma Cells and Overcomes Bortezomib Resistance. Cancer Cell. (2012) 22:345–58. doi: 10.1016/j.ccr.2012.08.007 PMC3478134 22975377

[11] De S Holvey-Bates EG Mahen K Willard B Stark GR. The ubiquitin E3 ligase FBXO22 degrades PD-L1 and sensitizes cancer cells to DNA damage. Proc Natl Acad Sci USA. (2021) 118:e2112674118. doi: 10.1073/pnas.2112674118 34795058 PMC8617495

[12] Qin L Song Y Zhang F Wang R Zhou L Jin S. CRL4B complex-mediated H2AK119 monoubiquitination restrains Th1 and Th2 cell differentiation. Cell Death Differ. (2023) 30:1488–502. doi: 10.1038/s41418-023-01155-8 PMC10244459 37024604

[13] Malarvannan M Unnikrishnan S Monohar S Ravichandiran V Paul D. Design and optimization strategies of PROTACs and its Application, Comparisons to other targeted protein degradation for multiple oncology therapies. Bioorg Chem. (2025) 154:107984. doi: 10.1016/j.bioorg.2024.107984 39591691

